# Dataset on Bac settlement stakeholders' perspectives on integrative urban design play as an instrument for managing wicked urban regeneration issues in sustainable way

**DOI:** 10.1016/j.dib.2023.109562

**Published:** 2023-09-16

**Authors:** Tatjana Mrdjenovic

**Affiliations:** University of Belgrade – Faculty of Architecture, Bulevar kralja Aleksandra 73/2, 11000 Belgrade, Serbia

**Keywords:** Participation, Communicative planning, Sustainable urban design, Urban regeneration, Protected urban areas

## Abstract

The presented data provides insights into the perspectives of influential stakeholders within the Bac community in Serbia regarding sustainable urban regeneration, urban design, and the role of integrative urban design. The data was collected subsequent to a collaborative workshop held in 2010, titled ``Integrative Urban Design Play (IUP) for Urban Regeneration on Bac Suburbia.'' During this workshop, attendees acquired novel insights into participatory approaches for fostering sustainable urban regeneration. The objective of the survey was to assess the perspectives of the different stakeholders regarding the feasibility of introducing novel urban development approaches in areas that are under conservation protection.

The intent of the research was to evaluate the effectiveness of a learning by doing approach in the implementation of new methods in urban development practise, with a focus on supporting broader concepts such as sustainability and urban regeneration. The research was designed to explore the varying perspectives of relevant stakeholders based on their profession to examine the potential for their clustering towards integrating diverse opinions into a broader understanding using IUP.

The efficacy of the IUP in addressing wicked urban regeneration problems, particularly in raising public awareness about the preservation, introduction, and promotion of essential values in protected suburban regions, was assessed through the administration of questionnaires. The questions were organised into three distinct categories: viewpoints on sustainable urban regeneration, perspectives on urban design, and perspectives on knowledge and readiness for future engagement in the Integrative Urban Design Game. A collaborative effort was undertaken by the Faculty of Architecture in Belgrade and the Municipality of Bac, Serbia, to administer an anonymous survey. This survey was disseminated to pertinent stakeholders representing the public sector.

The participants in the research exhibited diversity in terms of their occupation, level of experience, and age range, which spanned from 30 to 60 years old. They were chosen from among the attendees of the workshop based on their demonstrated interest, whether overt or covert, in addressing the complex issue at hand. A total of 28.57 percent of the participants were employed in the economic sector, specifically in tourism or management. Similarly, 14.28 percent of the examinees worked in the field of environmental studies, focusing on energy efficiency or natural resource protection. Another 14.28 percent were engaged in the legal profession, while 21.42 percent were involved in media and cultural activities. Additionally, 7.14 percent of the examinees were dedicated to the preservation of cultural heritage.

The present paper presents findings that illustrate the level of stakeholder receptiveness towards the sustained utilization of integrative methodologies, such as IUP, within the realm of local planning practice subsequent to the acquisition of novel knowledge and skills via workshops and training sessions. This paper showcases the efficacy of employing participatory techniques and skills to modify urban development and planning practices, with the aim of fostering shared understanding and agreement on environmental values. Consequently, it provides evidence of the effectiveness of the implemented approach within the specific community, as well as its potential applicability in similar communities.

Specifications Table

**Table:** 

Subject	Social Sciences.
Specific subject area	Providing quantitative and qualitative data on stakeholders´ attitudes on adopting sustainable urban development techniques the dataset is in Planning and Development area.
Data format	Raw, Analysed.
Type of data	Table, Image, Chart, Graph, Figure.
Data collection	The data collected is quantitative in nature and included structured and opened questionnaires, distributed to relevant stakeholders in Bac community. The objective and purpose of the research was clearly presented to the participants as well as its anonymous character. Three categories of topics were used to group the questions: Viewpoints on sustainable urban regeneration; Perspectives on urban design; and Perspectives on knowledge and readiness for participation in the Integrative Urban Design Play in future. The data was analysed in relation to stakeholders´ profession using chart.
Data source location	Municipality of Bac, SERBIA
Data accessibility	Repository name: Mendeley Data Data identification number: DOI:10.17632/grcdmwryy6.1 Direct URL to data: https://data.mendeley.com/datasets/grcdmwryy6/1
Related research article	/

## 1 Value of the Data

• The data provides insight on the impact of participatory workshop and its methods in changing opinions on sustainable approaches in urban regeneration.
• The presented data can help policymakers to develop more inclusive policies for sustainable urban regeneration, settling conflicts at the very beginning.
• Other researchers can use the dataset to compare it to information collected in comparable studies conducted in other geographical areas or regions.
• Other academics and researchers can use the data to expand analysis using SEM in path modelling.

## 2 Data Description

**Fig. 1:**
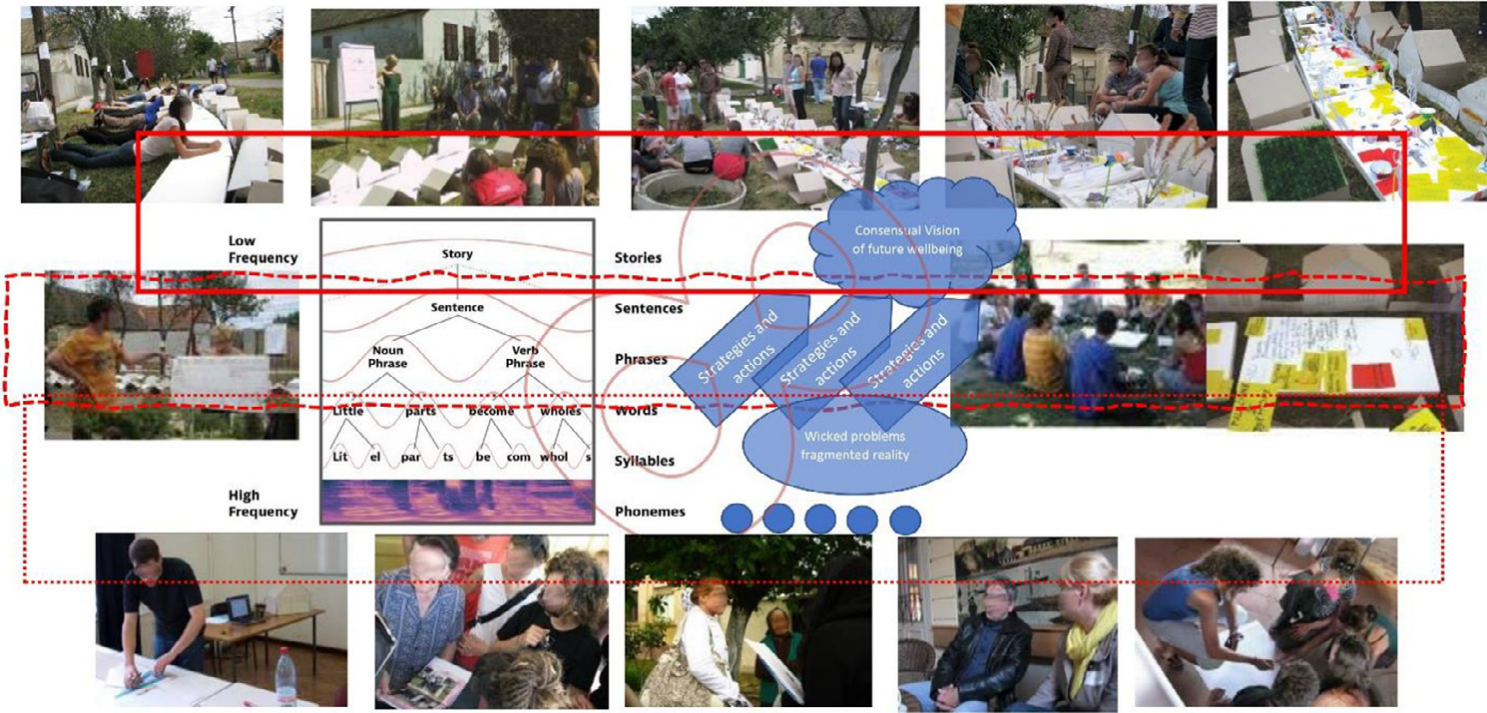
Main characteristics of the method Integrative Urban Design Play as communicative harmonization instrument in sustainable urban regeneration process.

Data presented in the paper represent opinions among stakeholders in Bač community on Sustainability, Urban regeneration, Integrative urban design and Integrative urban design play – IUP as a method for consensual communicative thinking in the process of sustainable urban regeneration [1], [2], [3], [4]. The degree of consensus (consistency) among viewpoints is utilised to identify discrepancies in opinions among various professional groups involved in the practise of sustainable urban regeneration. The gaps refer to areas that can be addressed through future capacity building workshops and partnership building initiatives. The measurement of consistency is conducted through the utilisation of primary data, which is presented in the initial table. This data is then visualised using a bubble chart, wherein professions that share similar opinions are positioned at the same level on the chart. Additionally, diagrams are employed to illustrate any gaps or trends in these opinions. The vast number of examinees were involved in the process of sustainable urban regeneration workshop which used method of Integrative Urban Design Play – IUP (Fig. 1). The outcomes derived from this initial workshop have been incorporated into the local urban development plans, specifically the detailed regulation plan for the Bac suburban area. Fig. 1. presents main characteristics of IUP from the point where its communicative harmonization potential is relevant in its critical phases: Analysis of present, Visioning and Strategizing in participative manner.

Fig. 1 represents Integrative Urban Design Play - IUP which refers to the holistic approach of planning and designing urban spaces. It involves the integration of various elements, such as architecture, landscape, transportation, activities, etc. The process of IUP can be understood as a form of harmonisation, drawing upon the ideas of Kant, Hekkert, Alexander, and Confucius, as well as the notion of harmony characterised by nested synchrony [5], [6], [7]. The utilisation of a mimetic framework in this context serves as a representation and educational instrument, symbolising a polygonal structure that facilitates the transition of a novel narrative pertaining to a particular urban environment. This transition is aimed at fostering a collective vision for the future, centred around the promotion of common welfare. Similar to social mimicry, the mimicry model within a particular urban context seeks to facilitate the survival and dissemination of culture by observing and imitating the behaviours of others. This model aims to transmit and cultivate a distinct local culture, encompassing its historical, contemporary, and future aspirations, while transcending physical and temporal limitations. Mimicry is regarded as a cognitive process through which knowledge can be transmitted intergenerationally, thereby circumventing the limitations of mimicry as a mere desire-driven behaviour. This transmission occurs via communicative actions, enabling the transfer of acquired knowledge from previous to subsequent generations. The phenomenon of mimicry enhances the conceptual framework of play, thereby engendering a novel narrative setting within its inherent ecological context.

The data presented in this table depicts the responses of stakeholders in the Bac community regarding the concept of sustainability. It encompasses their understanding of sustainability, the extent to which it is implemented in Bač, and the factors influencing the level of implementation. The data holds significant value as it demonstrates the correlation between the level of knowledge among examinees regarding sustainability and the preparedness of community administration to effectively implement sustainable practises [8].

The chart displays data pertaining to the clustering potential of professions within the Bač community, as determined by their perspectives on sustainability. According to a survey conducted among economists, 75% of respondents perceive sustainability as the integrative management of four pillars of sustainability, while the remaining 25% view it as the preservation and responsible utilisation of natural resources. Lawyers unanimously believe that the integration of the four pillars of sustainability, namely the economy, environment, society, and institutions, is of utmost importance. Similarly, this observation can be extended to include heritage protectors, media outlets, and other relevant entities. Approximately 50% of environmentalists align with the aforementioned category, while the remaining 50% perceive environmentalism as a conscientious approach towards the preservation and sustainable utilisation of natural resources. The perception among examinees is that the sustainability concept is only partially implemented in the Bac community. Specifically, 40% of economists attribute this partial implementation to a lack of knowledge and appropriate methods. Similarly, 50% of lawyers hold the same viewpoint. In contrast, all other professions unanimously believe that the partial implementation is solely due to a lack of adequate knowledge [8].

The data presented in this table depicts the responses of stakeholders in the Bac community regarding sustainable urban regeneration, including its definition, the extent of its implementation in Bač, and the factors influencing the current level of implementation. The data possesses significant value as it demonstrates the correlation between the knowledge levels of examinees regarding sustainable urban regeneration and the preparedness of community administration to effectively implement such practises. This correlation highlights the importance of education and awareness campaigns to increase knowledge among stakeholders and ensure successful implementation of sustainable urban regeneration in Bač. Additionally, the data can be used to identify specific areas where further support and resources are needed to overcome barriers and enhance the community's readiness for sustainable urban regeneration initiatives [9], [10], [11].

The statistics in this graphic illustrate the possibility of clustering stakeholders in the Bac community based on their views on sustainable urban regeneration. Only economists and media and culture stakeholders in 100% believe that urban regeneration is a process of economic, ecological, physical, ecological, and institutional development towards betterment in urban spaces and places. While 50% of lawyers believe the same, the other 50% believe that sustainable urban regeneration is huddling new activities into physically reconstructed areas, which is true for 100% of environmentalists, civil engineers, and heritage protectors. None of the examinees think that urban regeneration entails demolishing existing structures and erecting new ones. Despite their awareness of the concept, the majority of stakeholders believe that a lack of sufficient implementation methods hinders the execution of the idea of sustainable urban regeneration. They believe that urban regeneration should focus on preserving the cultural and historical significance of the existing structures while incorporating modern amenities and infrastructure. However, some stakeholders also express concerns about the potential displacement of local communities and the need for inclusive planning processes to ensure equitable development [8].

The chart provided herein presents the valuable feedback received from stakeholders within the Bac community regarding urban design. The responses cover a range of topics, including the definition of urban design, its relationship to urban regeneration and participation, the responsibilities of urban designers, and the outcomes that are produced. The stakeholders' responses showcase the wide range of perspectives on urban design within the Bac community. Different stakeholders have varying perspectives on urban design. Some view it as the art of crafting urban spaces that are both functional and aesthetically pleasing, while others place greater emphasis on its contribution to sustainability and liveability. Furthermore, the responses provide valuable insights into the potential of urban design to contribute to urban regeneration efforts, including the revitalization of neglected areas and the promotion of socio-economic growth [8].

The chart indicates that there is a strong consensus among respondents regarding the importance of urban design. A significant majority, 85% of participants, believe that urban design should be approached as a strategic process that integrates and involves various stakeholders. Furthermore, there is a shared belief that urban designers should play a facilitating role in this process. While there may be differing opinions on urban design output, it is widely recognised among professionals that it is preferable to approach it as an urban design framework or set of guidelines, rather than a rigid blueprint. There is a widely held belief that urban design should align with and reflect the goals and priorities outlined in higher-level plans and policies [8].

The chart presents data indicating a notable level of agreement regarding the connection between urban design and participation. While it is true that there is a difference in opinion among professionals regarding the level of community participation in the urban design process, it is worth noting that heritage protectors tend to prioritise the involvement of stakeholders from the public sector. According to a significant portion of economists, approximately 25%, there is a shared perspective. Similarly, a majority, around 66%, of stakeholders in the media and cultural sector hold a similar viewpoint. This indicates that there is a considerable consensus among these groups regarding the importance of community participation in urban design. However, it is important to acknowledge that there are still some professionals who may have differing opinions on the level of involvement required from the public sector in the urban design process [8].

The chart provided above displays the level of acceptance and success of a novel method known as Integrative Urban Design Play - IUP among stakeholders in the Bac community. This method has been utilised to address wicked urban regeneration problems in a sustainable manner. The method's acceptance and success were evaluated by asking questions about its key features, important advantages, and potential challenges for implementation. A significant majority of examinees, approximately 85%, perceive IUP as a highly effective approach to urban development. This is primarily due to its ability to foster a shared vision for urban regeneration, facilitate the gathering of diverse ideas, and establish a sustainable framework for regenerative processes. Additionally, the method's emphasis on stakeholder engagement and collaboration was seen as a crucial factor in its success. By involving various community members, including residents, potential investors, and local organisations, IUP ensures that all perspectives are considered and that decisions are made collectively. This inclusive approach not only increases the likelihood of successful implementation but also fosters a sense of ownership and pride among stakeholders, leading to long-term sustainability in urban development efforts [8].

According to the findings, 50 percent of economists see 'Integrative Urban Design Play' and its process as integrative in terms of several areas of sustainable development (economy, society, and environment). The other half perceive it as aesthetic, spatial-technical, and rich in regeneration options. Heritage protectors perceive it as completely integrative in terms of several areas of sustainable urban regeneration. Lawyers completely see it as strategic planning. The same is true for civil engineers, other professionals, and environmentalists who provide a spatial-technical component. According to 66.66 percent of media and cultural professionals, it is strategic planning, and 33.34 percent believe it is integrative in terms of multiple sectors of urban regeneration, as well as rich in regeneration alternatives [8].

Due to their belief in the effectiveness of the technique in urban regeneration in Bac settlement compared to traditional planning and design methods, a significant majority of 78.57 percent of respondents expressed their willingness to engage more actively in future processes. Approximately 7.14 percent of individuals hold a viewpoint that is not entirely positive, while around 14.28 percent remain undecided. It is widely acknowledged that the method has several positive aspects. These include fostering a shared vision for urban regeneration, generating a diverse range of ideas for potential solutions, fostering trust among different stakeholders to strengthen community bonds, establishing partnerships for effective implementation, and promoting the development of an urban design framework that prioritises the cultivation of social capital for enhanced harmony [8].

The chart presents the responses of various professional groups to specific survey questions, reflecting their respective opinions. The questions are clearly presented within the table featured in the subsequent chapter. The chart accurately represents the data that is presented in the original table, which serves as the primary source of data [8].

## 3 Experimental Design, Materials and Methods

**Chart 1:**
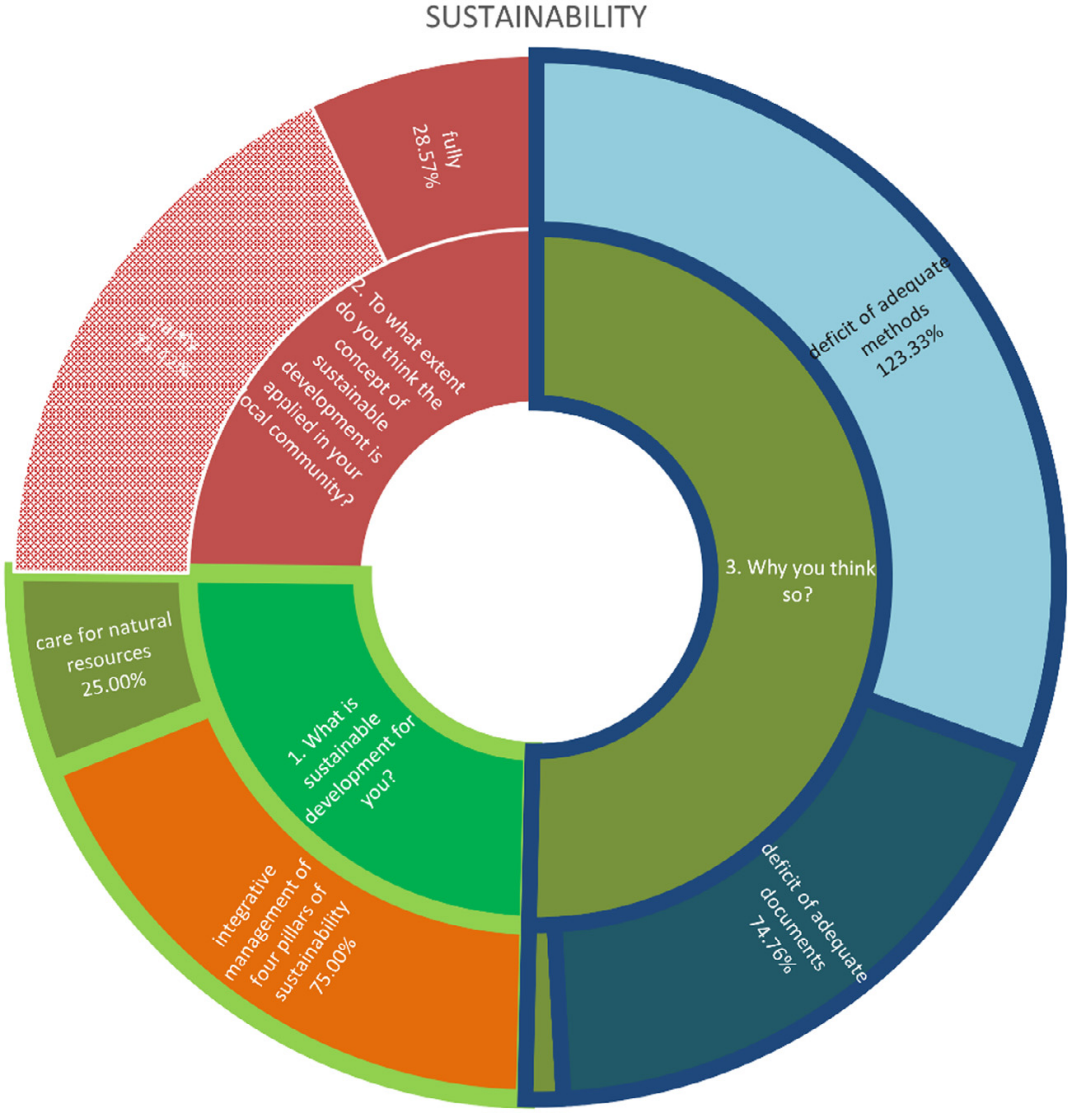
Opinions among stakeholders on sustainability.

**Chart 2:**
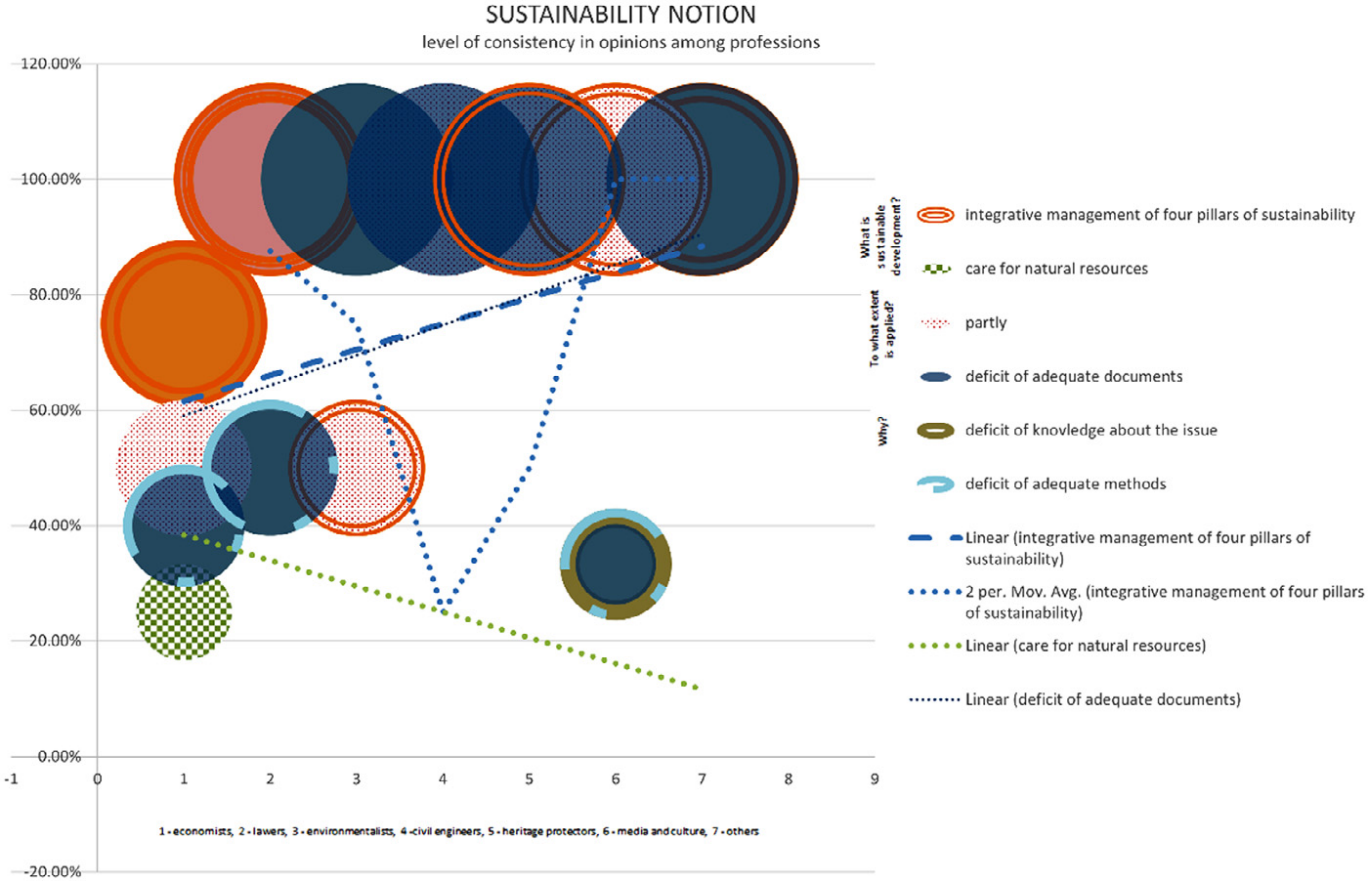
Level of consistency in opinions among professions in Bač community on sustainability.

**Chart 3:**
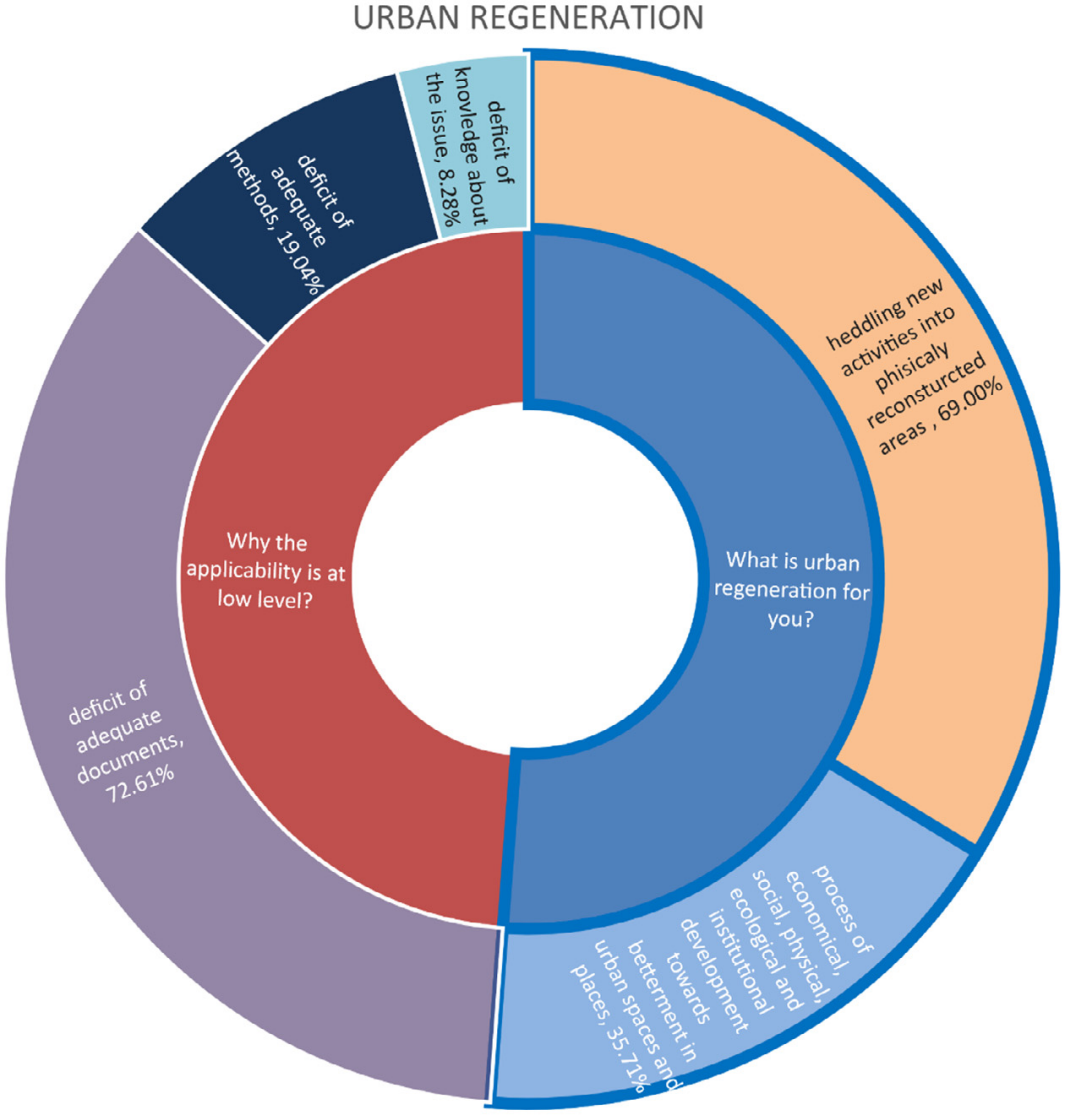
Opinions among stakeholders on Urban regeneration.

**Chart 4:**
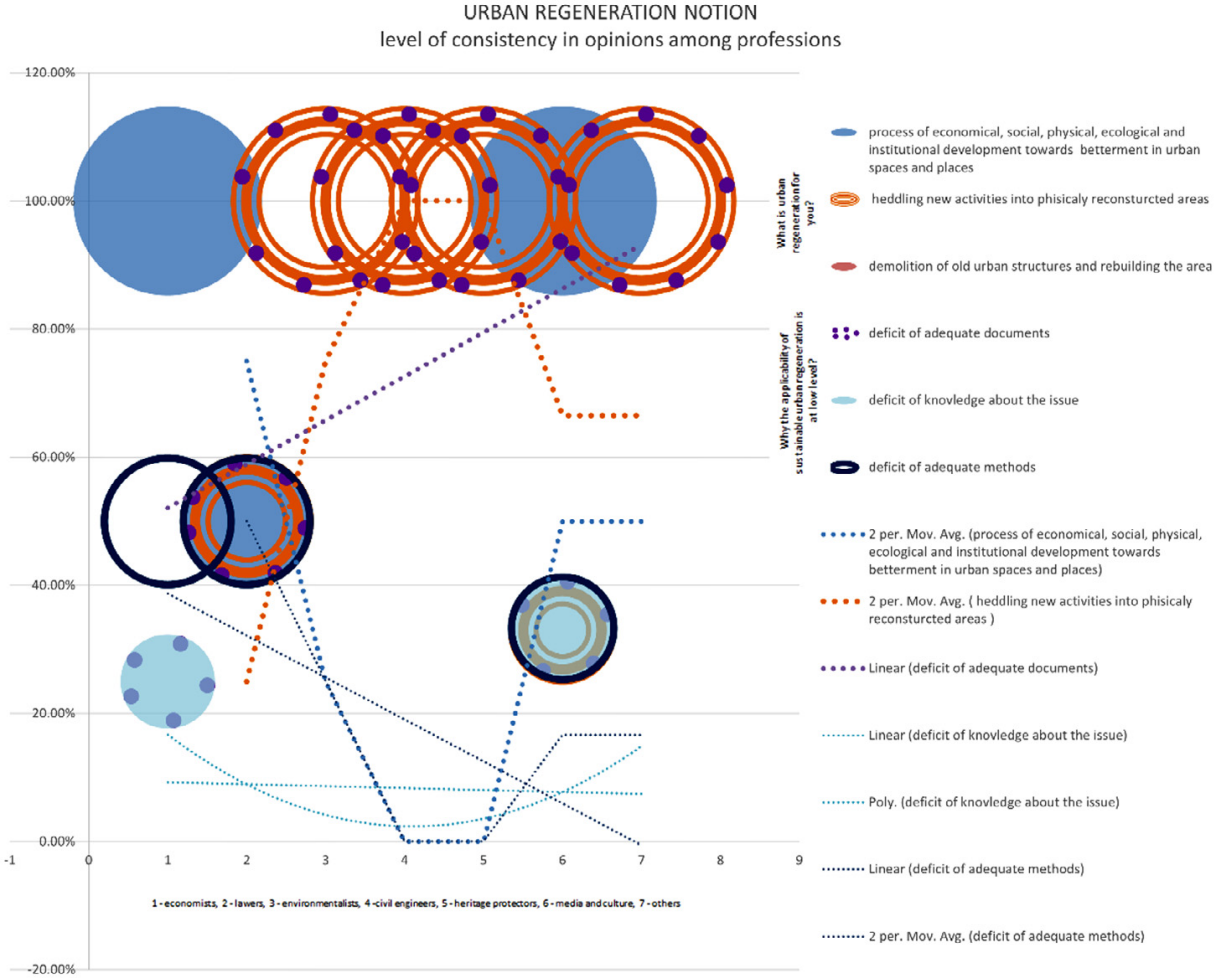
Level of consistency in opinions among professions in Bač community on urban regeneration.

**Chart 5:**
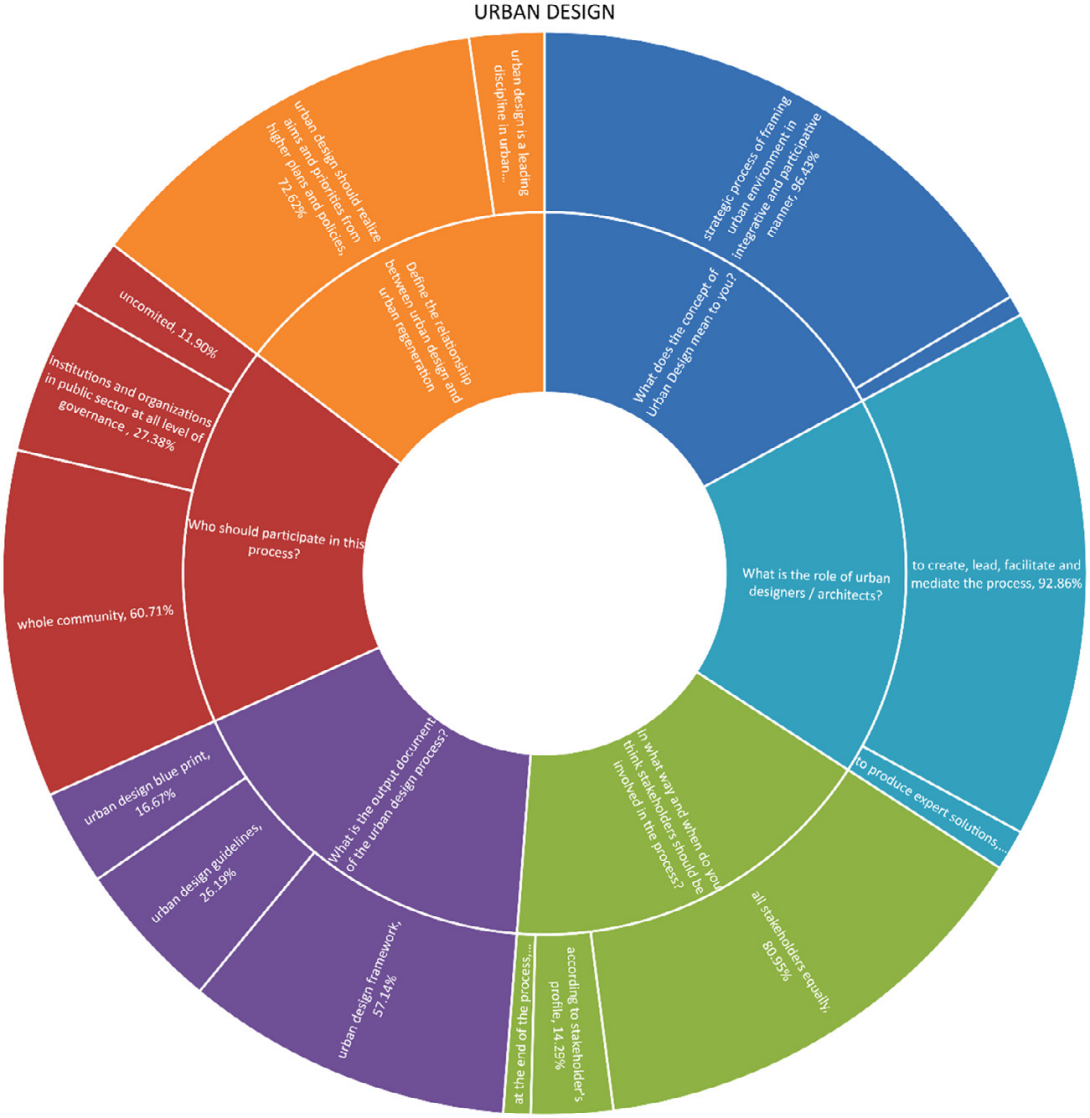
Opinions among stakeholders on Urban design.

**Chart 6:**
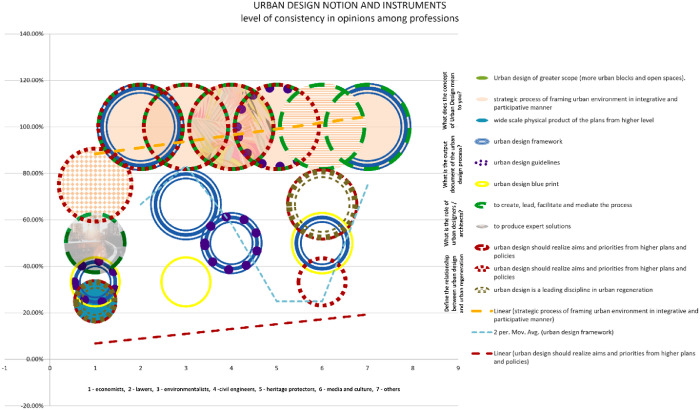
Level of consistency in opinions among professions in Bač community on Urban design.

**Chart 7:**
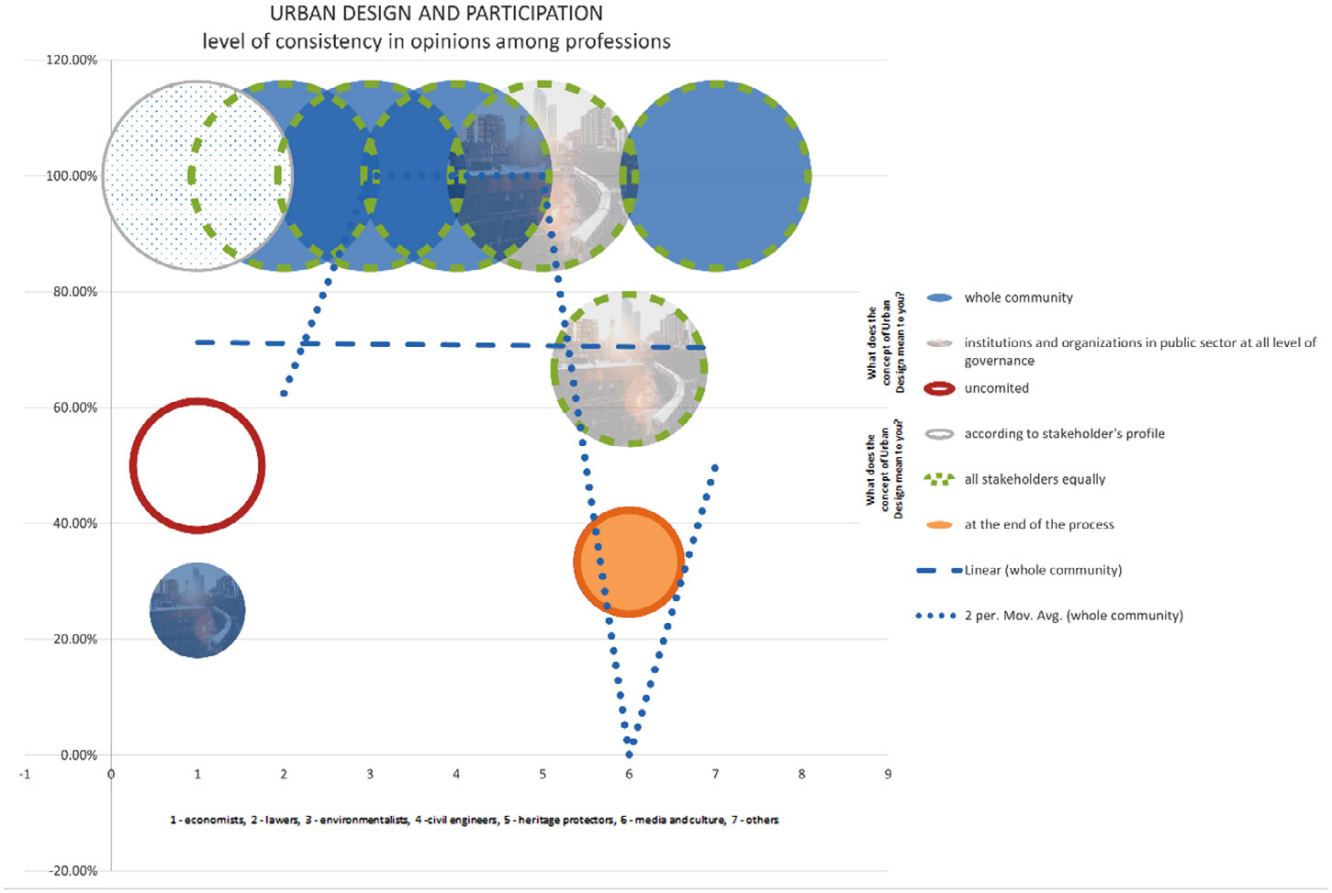
Level of consistency in opinions among professions in Bač community on Urban design and participation.

**Chart 8:**
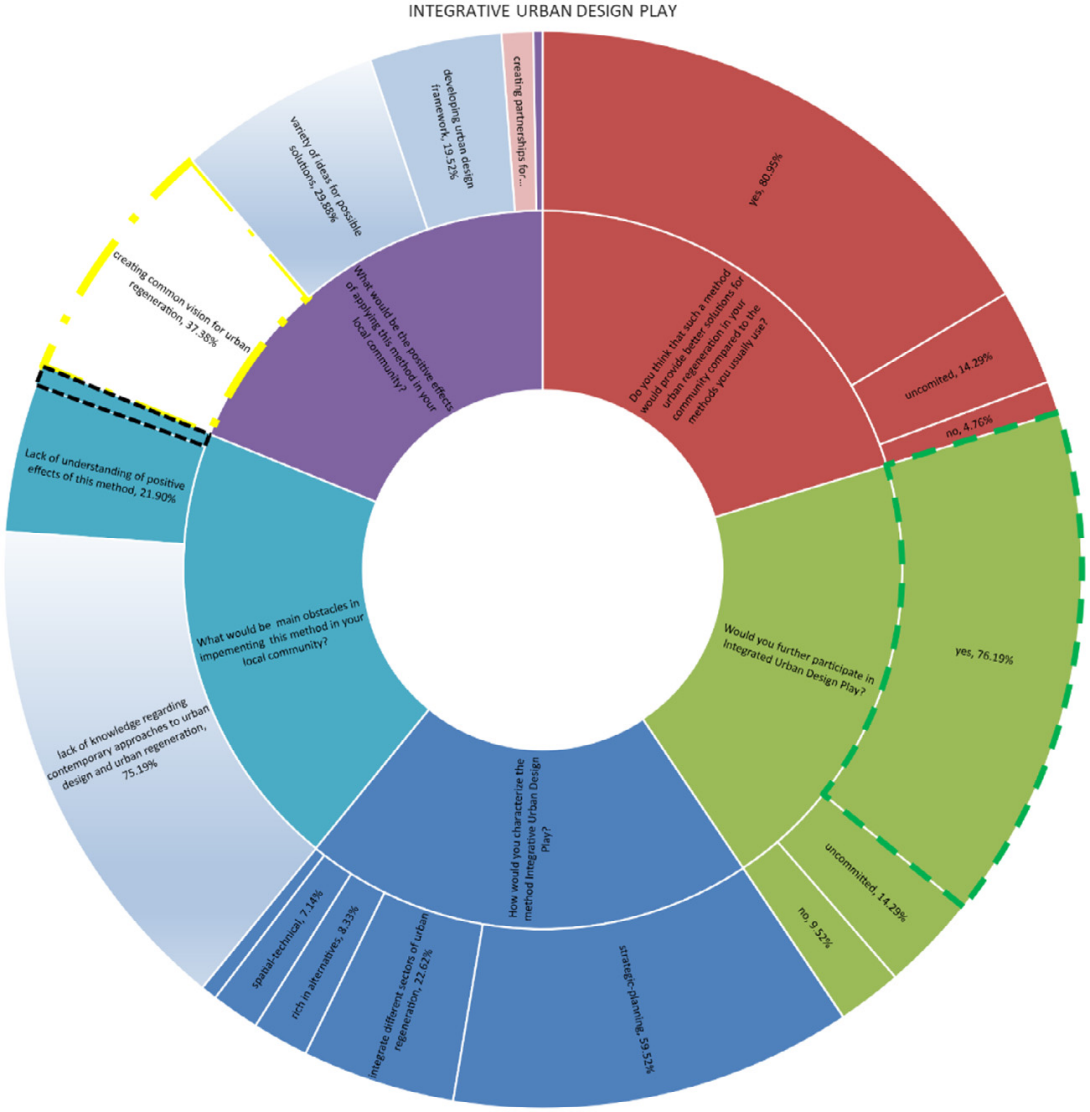
Opinions among stakeholders on IUP – Integrative urban design play.

**Chart 9:**
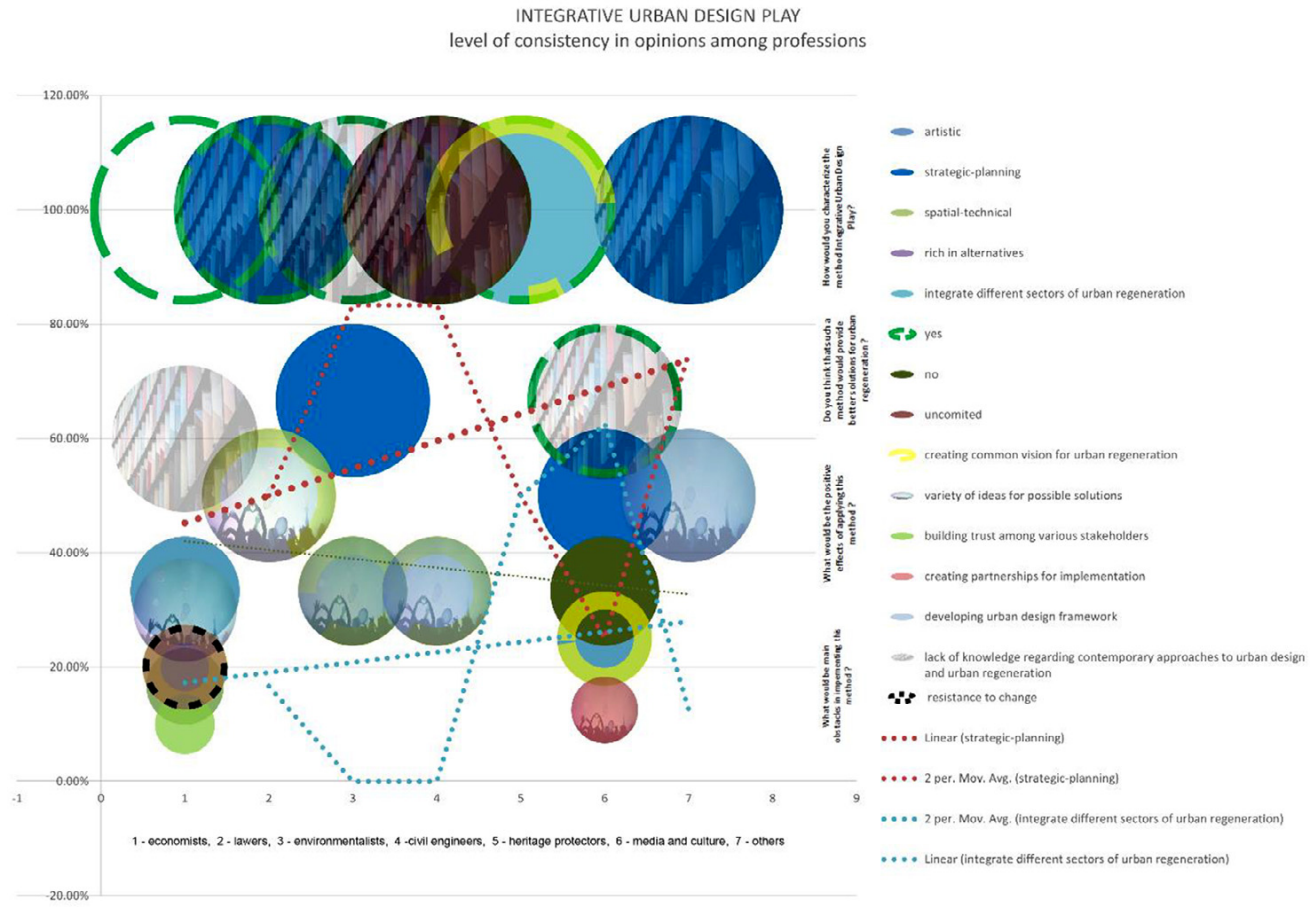
Level of consistency in opinions among professions in Bač community on IUP – Integrative urban design play.

**Chart 10:**
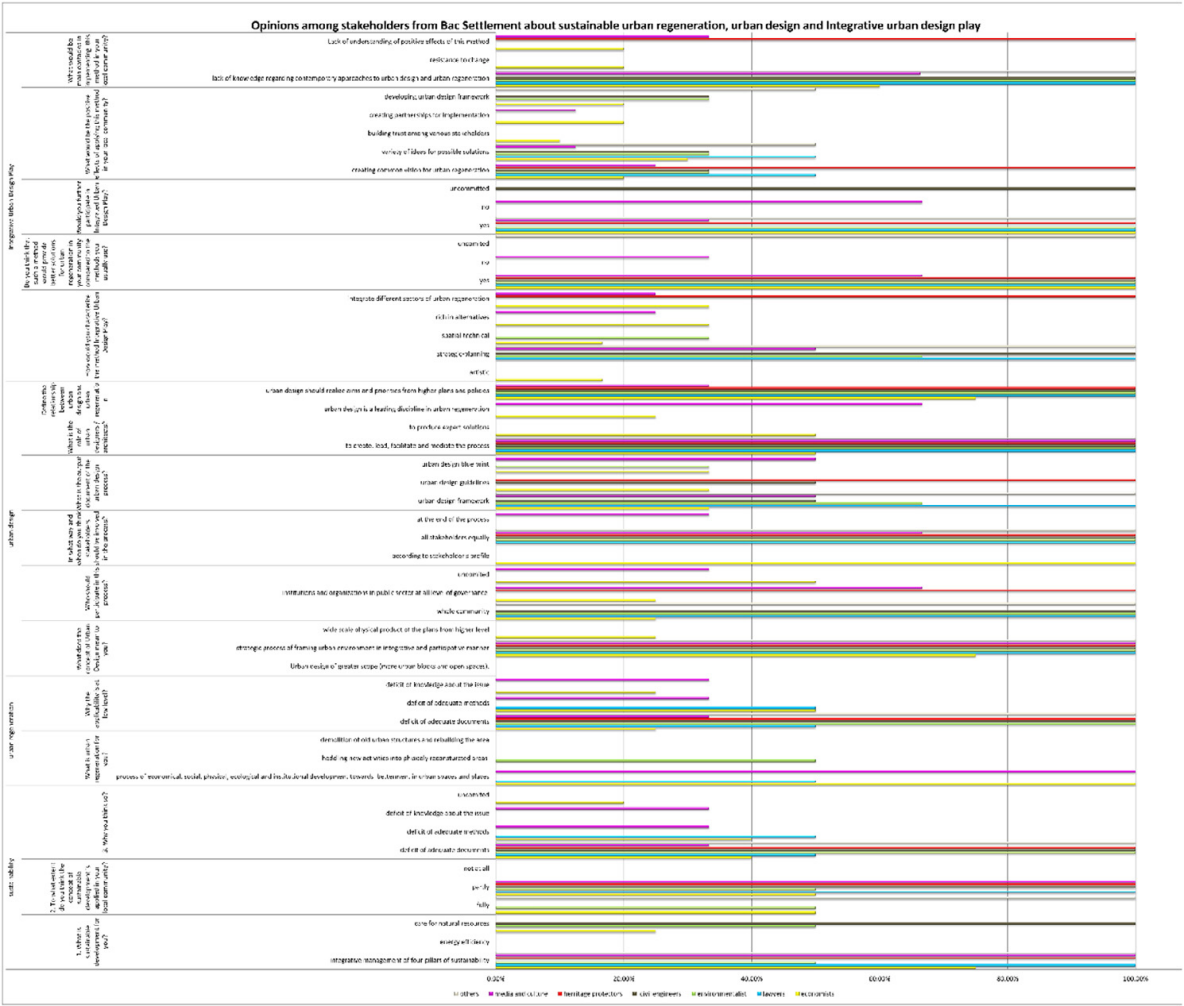
Presentation of opinions in percentages by profession.

The questionnaires, which included both closed and open-ended questions, were distributed to key members of the Bac community in order to collect quantitative and qualitative data. Participants were provided with clear and comprehensive information regarding the objectives and intentions of the research, as well as the assurance of their own confidentiality. The questions were thoughtfully categorized into three groups, each focusing on different subjects: sustainability, urban regeneration, and urban design, Integrative Urban Design Play (Table 1) (Chart 1, Chart 2, Chart 3, Chart 4, Chart 5, Chart 6, Chart 7, Chart 8, Chart 9, Chart 10).

**Table 1:** The research employs a question-and-answer format, with the results being presented in tabular form.

sustainability	1. What is sustainable development for you?	integrative management of four pillars of sustainability
		energy efficiency
		care for natural resources
	2. To what extent do you think the concept of sustainable development is applied in your local community?	fully
		partly
		not at all
	3. Why you think so?	deficit of adequate documents
		deficit of adequate methods
		deficit of knowledge about the issue
		uncommitted
urban regeneration	What is urban regeneration for you?	process of economical, social, physical, ecological and institutional development towards betterment in urban spaces and places
		huddling new activities into physically reconstructed areas
		demolition of old urban structures and rebuilding the area
	Why the applicability is at low level?	deficit of adequate documents
		deficit of adequate methods
		deficit of knowledge about the issue
urban design	What does the concept of Urban Design mean to you?	Urban design of greater scope (more urban blocks and open spaces).
		strategic process of framing urban environment in integrative and participative manner
		wide scale physical product of the plans from higher level
	Who should participate in this process?	whole community
		institutions and organizations in public sector at all level of governance
		uncommitted
	In what way and when do you think stakeholders should be involved in the process?	according to stakeholder's profile
		all stakeholders equally
		at the end of the process
	What is the output document of the urban design process?	urban design framework
		urban design guidelines
		urban design blue print
	What is the role of urban designers / architects?	to create, lead, facilitate and mediate the process
		to produce expert solutions
	Define the relationship between urban design and urban regeneration	urban design is a leading discipline in urban regeneration
		urban design should realize aims and priorities from higher plans and policies
Integrative Urban Design Play	How would you characterize the method Integrative Urban Design Play?	artistic
		strategic-planning
		spatial-technical
		rich in alternatives
		integrate different sectors of urban regeneration
	Do you think that such a method would provide better solutions for urban regeneration in your community compared to the methods you usually use?	yes
		no
		uncommitted
	Would you further participate in Integrated Urban Design Play?	yes
		no
		uncommitted
	What would be the positive effects of applying this method in your local community?	creating common vision for urban regeneration
		variety of ideas for possible solutions
		building trust among various stakeholders
		creating partnerships for implementation
		developing urban design framework
	What would be main obstacles in implementing this method in your local community?	lack of knowledge regarding contemporary approaches to urban design and urban regeneration
		resistance to change
		Lack of understanding of positive effects of this method

## Limitations

None.

## Ethics Statement

**Data collected in the research through questionnaire was** obtained from participants whose data has been fully anonymized. The questionnaire was designed to respect dignity, integrity, right to self-determination, privacy, and confidentiality of personal information of research subjects.

## CRediT authorship contribution statement

**Tatjana Mrdjenovic:** Conceptualization, Methodology, Visualization, Investigation, Writing – original draft.

## Data Availability

Opinions among stakeholders from Bac Settlement about sustainable urban regeneration, urban design and Integrative urban design play (Original data) (Mendeley Data)
